# Seroprevalence of six pathogens transmitted by the *Ixodes ricinus* ticks in asymptomatic individuals with HIV infection and in blood donors

**DOI:** 10.1038/s41598-019-38755-9

**Published:** 2019-02-14

**Authors:** Agnieszka Pawełczyk, Małgorzata Bednarska, Justyna D. Kowalska, Beata Uszyńska-Kałuża, Marek Radkowski, Renata Welc-Falęciak

**Affiliations:** 1 Department of Immunopathology of Infectious and Parasitic Diseases, Medical University of Warsaw, 3C Pawińskiego Street, 02-106 Warsaw, Poland; 2 Department of Parasitology, Faculty of Biology, University of Warsaw, 1 Miecznikowa Street, 02-096 Warsaw, Poland; 3 Department of Adults’ Infectious Diseases, Medical University of Warsaw, 37 Wolska Street, 01-201 Warsaw, Poland; 4 Blood Center of the Ministry of Internal Affairs and Administration, 137 Wołoska Street, 02-507 Warsaw, Poland; 5 AmerLab Ltd. Diagnostic Laboratory of Parasitic Diseases and Zoonotic Infections, Biological and Chemical Research Centre, 101 Żwirki and Wigury Street, 02-089 Warsaw, Poland

## Abstract

The objective of our study was to estimate the seroprevalence of six pathogens transmitted by ticks in HIV-infected persons and blood donors in Poland (*B*. *burgdorferi* s.l., *A*. *phagocytophilum*, *Ehrlichia* spp., *Babesia* spp., *Rickettsia* spp. *Bartonella henselae*) to assess the frequency of exposure to such microorganisms in immunocompetent and immunocompromised individuals in endemic regions for *I*. *ricinus* ticks. Serum samples were collected from 227 HIV-infected patients and 199 blood donors. All samples were analyzed for antibodies against six tick-borne pathogens and seroprevalence rates were statistically compared between two tested group as well as age, sex and lymphocyte T CD4+ level in HIV infected patients. The seroprevalence of tick-borne infections in HIV-infected patients is higher than that of the healthy population in Poland, although no association between serological status of patients and lymphocyte CD4+ T cell level has been observed. The frequency of tick-borne coinfections and doubtful results of serological tests were significantly higher in HIV-positive individuals. In Poland, the possibility of tick-borne diseases transmission with blood is rather negligible.

## Introduction

Recently the experts of the Center for Disease Control and Prevention’s have summarized the alarming increase in the number of vector-borne disease cases reported in the United States and territories from 2004 to 2016^1^. Of the almost 650,000 cases, over 491,000 were tick-borne. However, tick-borne diseases are a large and growing public health problem not only in the United States but also in Europe^2^. *Ixodes ricinus* is the most widespread tick species in Europe and constitutes the vector of numerous pathogens, especially *Borrelia burgdorferi* sl. and *Borrelia myiamotoi*, *Anaplasma phagocytophilum*, tick-borne encephalitis virus (TBEV), *Babesia* spp., as well as some *Rickettsia* and *Ehrlichia* species^3–6^. Lyme borreliosis (LB) is the most common vector-borne disease in temperate zones of the northern hemisphere, and about 85,000 cases are reported annually in Europe^7^. The estimated incidence of LB in Poland increased dramatically from 20.3 per 100,000 inhabitants in 2007 to 56.0 per 100,000 inhabitants in 2017 (an estimated average increased from 7,735 cases per year in 2007 to 21,516 cases per year in 2017)^8^. As of today, there are about 100 confirmed or probable cases of anaplasmosis and about 60 cases of babesiosis in Europe^9,10^, including Poland^11–13^. The *Rickettsia* infections and single cases of human granulocytic ehrlichiosis (HGE) have been also noted in Europe^14,15^. Recent data suggest that ticks could also transmit *Bartonella henselae* to human^16–19^. Immunocompetent individuals with tick-borne infections may present with non-specific symptoms, such as fever and a flu-like disease which usually abate spontaneously within a few weeks^9,15,20,21^. Nevertheless, severe infections in immunocompetent humans have been also noted^22–24^. Furthermore, asymptomatic tick-borne infections in healthy persons may constitute threats to the safety of the blood supply^25,26^. However, in individuals with immunologically compromising conditions, including HIV-1 (human immunodeficiency virus type 1)-positive patients, tick-borne pathogens may cause chronic, debilitating opportunistic infection and even death^27–32^.

Patients diagnosed with HIV-1 are immunodeficient, which is a significant risk factor for diseases caused by specific pathogens, namely those expanding due to the lower level of T lymphocyte (LT) CD4+ cells, since pathogenicity often depends on cellular and humoral immune responses^33^. In Poland, since 1985, there have been about 22,000 new cases of HIV infection^8^. As the positive predictive value of serological tests is reduced, in HIV-positive patients diagnostics based on such methods used to be cumbersome^34^. Improvement in treatment efficacy has resulted in better immune system function of the majority of HIV-positive patients; another consequence has been a significant increase in the positive predictive value (PPV) risk of serology-based methods. Prognosis for patients with HIV-1 has improved pronouncedly since the commencement of HAART (highly active antiretroviral therapy) which involves both antiretroviral drugs and efficient regimens. Consequently, HIV-infected individuals have greater chance of living actively, yet engaging in outdoor activities is a risk factor for tick infestation^35,36^. Until now, there have been only a few studies concerning occurrence of tick-borne diseases in HIV-positive patients, in contrast to other infections associated with the same virus. Additionally, the studies that had been conducted were analyses of mainly single clinical cases, and only *Babesia*, *Borrelia*, *A*. *phagocytophilum* and *Rickettsia* have been detected in HIV-infected individuals in Europe so far, out of the broad spectrum of tick-borne pathogens^37–40^.

The objective of our study was to estimate the seroprevalence of six pathogens transmitted by ticks (*B*. *burgdorferi* s.l., *A*. *phagocytophilum*, *Ehrlichia* spp., *Babesia* spp., *Rickettsia* spp. *Bartonella henselae*) in HIV-infected persons and blood donors (representing the control group) in Poland, to assess the frequency of exposure to such microorganisms in immunocompetent and immunocompromised individuals in endemic regions for *I*. *ricinus* ticks^41^. To the best of our knowledge, this is the first serological study on the occurrence of the most common pathogens transmitted by ticks in HIV-1-infected humans.

## Results

### Description of the tested group of patients/blood donors

Of the 227 HIV-infected patients included in the study, the medical data (lymphocyte CD4+ T cell level, plasma HIV RNA level, HAART therapy, age and sex, risk group [MSM, injection drug user]) were obtained from 148 patients. In this group, with the mean age of 33 years (range 20–51 years), men predominated (140 patients, 95%). The median lymphocyte CD4+ T cell count was 465/µl with 19% of patients with less than 300/µl. Most of these patients (82%; n = 121) were on HAART.

Of the 199 blood donors, 134 men and 65 women were included with the mean age of 36 (range 18–71 years). The majority of participants (95%; n = 190) inhabit urban areas, however, 32% of them (n = 63) declared contact with ticks, mainly in natural areas (forest). Lyme borreliosis was diagnosed and then treated in the last 10 years in 9 participants (4.5%).

### Borrelia burgdorferi s.l. seroprevalence

Of the 227 HIV-infected patients tested for *B*. *burgdorferi* s.l. specific IgM and IgG by ELISA test, 66 and 11 were positive, respectively, which corresponds to the seroprevalence rate of 29.1% for IgM and 4.8% for IgG (Table 1). Among 199 serum samples collected from blood donors, 26 (13.1%) for IgM and 10 (5.0%) for IgG were positive, respectively. The IgM seroprevalence noted in HIV-infected patients was significantly higher compared to seroprevalence observed in blood donors (29.1% vs. 13.1%; χ^2^ = 16.58, df = 1, p = 0.0001); however, IgG seroprevalence did not differ statistically between these two groups (Table 1).

**Table 1:** Prevalence of anti-*B*. *burgdorferi*, anti-*B*. *microti*, anti-*A*. *phagocytophilum*, anti-*Ehrlichia* spp., anti-*B*. *henselae* and anti-*Rickettsia* spp. IgM and IgG antibodies in HIV-infected patients and blood donors in Poland.

	Method	Tested group of patients	IgM			IgG		
			Positive (%)	Negative (%)	P	Positive (%)	Negative (%)	P
*Borrelia burgdorferi* s.l.	ELISA	HIV-positive	66/227 (29.1)	161/227 (70.9)	<10^−4^	11/227 (4.8)	216/227 (95.2)	0.932
		blood donors	26/199 (13.1)	26/199 (86.9)		10/199 (5.0)	189/199 (95.0)	
*Babesia microti*	IFA	HIV-positive	21/227 (9.3)	206/227 (90.7)	<10^−4^	5/227 (2.2)	222/227 (97.8)	0.595
		blood donors	2/199 (1.0)	197/199 (99.0)		3/199 (1.5)	196/199 (98.5)	
*Anaplasma phagocytophilum*	IFA	HIV-positive	6/277 (2.6)	221/227 (97.4)	0.817	6/227 (2.6)	221/227 (97.4)	0.410
		blood donors	6/199 (3.0)	193/199 (97.0)		3/199 (1.5)	196/199 (98.5)	
*Ehrlichia* spp.	IFA	HIV-positive	4/227 (1.8)	223/227 (98.2)	0.096	16/227 (7.0)	211/227 (93.0)	0.026
		blood donors	9/199 (4.5)	190/199 (95.5)		5/199 (2.5)	194/199 (97.5)	
*Bartonella henselae*	IFA	HIV-positive	10/227 (4.4)	217/227 (95.6)	0.953	4/227 (1.8)	223/227 (95.5)	0.096
		blood donors	9/199 (4.5)	190/199 (95.5)		9/199 (4.5)	190/199 (95.5)	
*Rickettsia* SFG	ELISA	HIV-positive	nd	nd	nd	4/227 (1.8)	223/227 (98.2)	0.503
		blood donors	nd	nd		2/199 (1.0)	197/199 (99.0)	

All positive results of ELISA tests were confirmed by Western Blot. Out of the IgM and IgG ELISA-positive samples of HIV infected patients, half were confirmed in WB (50% [33/66] and 53.8% [6/11] for IgM and IgG, respectively). Statistically lower IgM ELISA positive results were confirmed in WB in blood donors (23.1% [6/26]; χ^2^ = 4.49, df = 1, p = 0.034). However, of the 10 IgG ELISA positive results, 8 (80%) were confirmed in WB (statistically not significant).

**Figure 1:**
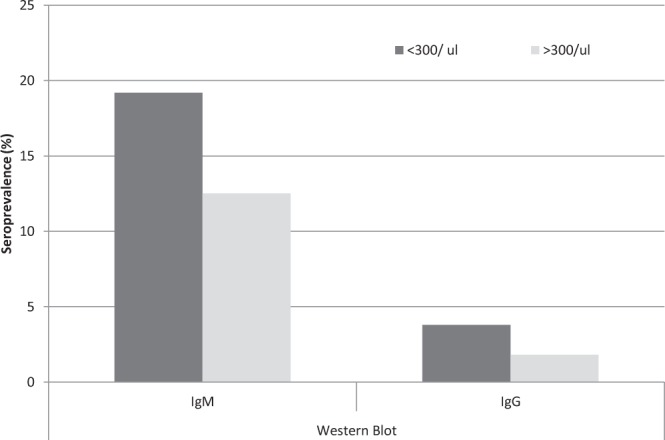
Seroprevalence of anti-*Borrelia burgdorferi* s.l. antibodies in HIV-infected patients with median lymphocyte CD4+ T cell count <or >300/μl.

Due to a sufficient number of *B*. *burgdorferi* seropositive cases in WB for HIV-positive patients, the effect of sex, age and lymphocyte CD4+ T cell level on seroprevalence were estimated. IgM seroprevalence increased significantly with age, reaching a maximum of 36.8% for patients aged >35 years (<25 years: 0%; 25–35 years: 11.1%; χ^2^ = 7.84, df = 2, p = 0.0001). IgG seroprevalence noted for patients aged 25–35 years was almost twice as low as in patients aged >35 years (2.8% vs. 5.3%; no statistical differences). No statistical difference of seroprevalence between men and women was observed (IgM: 15.3% vs 10.0%; IgG: 2.8% vs. 0.0%, respectively). *Borrelia* IgM and IgG seroprevalence were higher in HIV-infected patients with lymphocyte CD4+ T cell count less than 300/µl (IgM: 19.2% vs. 12.5%; IgG: 3.8% vs. 1.8%, no statistical differences) (Fig. 1).

### Babesia microti seroprevalence

The IgM seroprevalence was about 9 times higher in HIV-infected patients (9.3%; 21/227) than in blood donors (1.0%; 2/199) (χ2 = 16.65, df = 1, p = 0.0001) (Table 1). *Babesia* IgG seroprevalence did not differ statistically between HIV-positive patients and blood donors (2.2% [5/227] vs. 1.5% [3/199]). No significant association was demonstrated between the serological status of the tested group of patients/participants and age, sex or lymphocyte CD4+ T cell level in HIV-positive patients.

### Anaplasma phagocytophilum seroprevalence

The IgM and IgG seroprevalence was similar in HIV-infected patients and blood donors (IgM: 2.6% [6/227] vs. 3.0% [6/199], IgG: 2.6% [6/227] vs. 1.5% [3/199], respectively) (Table 1). No significant association was demonstrated between the serological status of the tested group of patients/participants and age, sex or lymphocyte CD4+ T cell level in HIV-positive patients.

### Ehrlichia chaffeensis seroprevalence

Of the 227 HIV-positive patients tested for *Ehrlichia*, 4 (1.8%; 4/227) were positive for specific IgM (Table 1). The IgM seroprevalence in blood donors was 2.5 times higher (4.5%; 9/199), but this difference was not statistically significant. Nevertheless, IgG seroprevalence noted in HIV-infected patients was significantly higher compared to seroprevalence observed in blood donors (7.0% [16/227] vs. 2.5% [5/199]; χ^2^ = 4.93, df = 1, p = 0.026). No significant association was demonstrated between the serological status of the tested group of patients/participants and age, sex or lymphocyte CD4+ T cell level in HIV-positive patients.

### Bartonella henselae seroprevalence

The IgM and IgG seroprevalence was similar in HIV-infected patients and blood donors (IgM: 4.4% [10/227] vs. 4.5% [9/199], IgG: 1.8% [4/227] vs. 4.5% [9/199], respectively) (Table 1). No significant association was demonstrated between the serological status of the tested group of patients/participants and age, sex or lymphocyte CD4+ T cell level in HIV-positive patients.

### Rickettsia Spotted-Fever Group seroprevalence

The IgG seroprevalence for *Rickettsia* SFG did not differ significantly between HIV-positive patients and blood donors (1.8% [4/227] vs. 1.0% [2/199]). No significant association was demonstrated between the serological status of the tested group of patients/participants and age, sex or lymphocyte CD4+ T cell level in HIV-positive patients.

### Multiple seropositives

**Figure 2:**
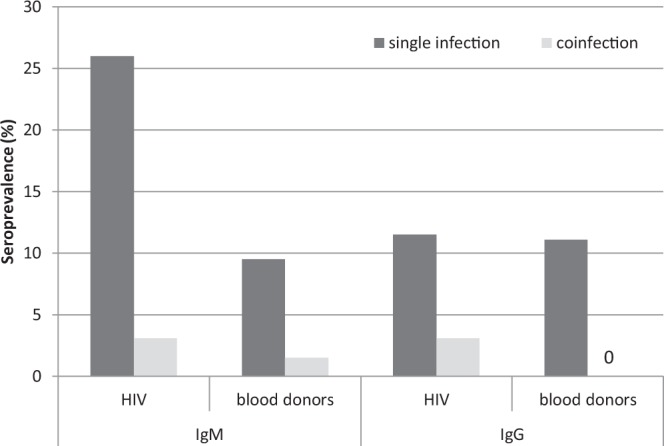
Seroprevalence of specific antibodies in single infection and coinfection with two pathogens in HIV-infected patients and blood donors in Poland.

Among 227 HIV-positive patients who were tested for six tick-borne pathogens, the IgM serology was positive for a single pathogen in 26.0% (59/227) and for two pathogens in 3.1% (7/227), especially *B*. *microti* together with *B*. *burgdorferi* (3/7) or *B*. *henselae* (2/7) and *A*. *phagocytophilum* with *B*. *henselae* (1/7) or *E*. *chaffeensis* (1/7) (Fig. 2). The IgM seroprevalence rate was significantly lower in blood donors in whom a single pathogen was noted in 9.5% (19/199) and two pathogens in 1.5% (3/199), in all cases *A*. *phagocytophilum* with *E*. *chaffeenisis* were involved (χ2 = 22.01, df = 2, p = 0.001).

The rate of IgG seroprevalence of a single pathogen was similar in HIV-positive patients and blood donors (11.5% [26/227] vs. 11.1% [22/199]). However, the infections of two pathogens were noted only in HIV-infected patients (3.1%; 7/227) (χ2 = 8.96, df = 2, p = 0.011): *A*. *phagocytophilum* with *E*. *chaffeenisis* (6/7) and one case of *Rickettsia* with *B*. *henselae* (Fig. 2). No significant association was demonstrated between the serological status of tested group of patients/participants and age, sex or lymphocyte CD4+ T cell level in HIV-positive patients.

### Doubtful results

The frequency of doubtful results was significantly higher in HIV-infected patients than in blood donors (Table 2). In HIV-positive patients the doubtful results for *A*. *phagocytophilum* were 9.7% and 6.2%, for *E*. *chaffeensis-* 4.4% and 6.6%, for *B*. *henselae*- 2.6% and 2.2% for IgM and IgG tests, respectively, whereas the doubtful results for these pathogens were not noted in blood donors. For *B*. *burgdorferi* IgM ELISA test, the amount of doubtful results was similar in both tested groups of patients/participants. In HIV-positive patients, the doubtful results for *B*. *burgdorferi* IgG ELISA test were observed more often (2.6% vs. 0.5%); however, this difference was not statistically significant.

**Table 2:** The frequency of doubtful results in HIV-infected patients and blood donors in Poland.

	Method	Tested group of patients	IgM		IgG	
			Doubtful (%)	P	Doubtful (%)	P
*Borrelia burgdorferi* s.l.	ELISA	HIV-positive	18/227 (7.9)	0.555	6/227 (2.6)	0.066
		blood donors	19/199 (9.5)		1/199 (0.5)	
*Babesia microti*	IFA	HIV-positive	0/227 (0.0)	0.080	0/227 (0.0)	1.000
		blood donors	2/199 (1.0)		0/199 (0.0)	
*Anaplasma phagocytophilum*	IFA	HIV-positive	22/277 (9.7)	<10^−4^	14/277 (6.2)	<10^−4^
		blood donors	0/199 (0.0)		0/199 (0.0)	
*Ehrlichia* spp.	IFA	HIV-positive	10/227 (4.4)	<10^−4^	15/227 (6.6)	<10^−4^
		blood donors	0/199 (0.0)		0/199 (0.0)	
*Bartonella henselae*	IFA	HIV-positive	6/227 (2.6)	0.006	5/227 (2.2)	0.012
		blood donors	0/199 (0.0)		0/199 (0.0)	
*Rickettsia* SFG	ELISA	HIV-positive	nd	nd	0/227 (0.0)	1.000
		blood donors	nd		0/199 (0.0)	

## Discussion

The large population of immunocompromised patients, including those with HIV/AIDS, is constantly growing. Analysis of the published reports of tick-borne infections shows that the disease in immunocompromised patients is far more severe, prolonged and more likely to be fatal^9,14,28^. Thus far, investigations of the epidemiology of tick-borne pathogen infections involving serological studies have concentrated on inhabitants of endemic regions who were healthy and whose immunological function was normal. By contrast to those studies or to reviews of clinical findings concerning hospitalized patients, our molecular and retrospective study had a different objective: establishing both the incidence and seroprevalence of tick-borne infections in patients with HIV, compared with the control group of healthy blood donors without clinical symptoms. Both groups were based in an endemic area of *I*. *ricinus* ticks, namely Poland. The Polish Society of Epidemiology and Infectious Diseases has issued a recommendation regarding diagnostics of tick-borne diseases to which this study particularly conforms.

In HIV-positive patients, borreliosis is rarely reported as co-infection, with only a few cases so far^28,42^. Most of them were identified early, with neuroborreliosis confirmed in Dutch and Swedish patients. Our recent molecular study confirmed the asymptomatic *B*. *garinii* infection in an HIV-postitive patient with no signs of early or late-stage Lyme borreliosis^40^. *Borrelia burgdorferi* was the predominant pathogen in our study, and about 30% and 5% of HIV-infected individuals were recorded as positive for IgM and IgG, respectively. In this group of patients, the IgM seroprevalence rate was significantly higher compared to blood donors. The significantly higher *B*. *burgdorferi* seroprevalence was observed in HIV-infected patients aged >35 years and with a median lymphocyte CD4+ T cell less than 300/μl. To the best of our knowledge, as of today, only one serological study of *Borrelia* infection in HIV-postitive patients was conducted, and the results were comparable – a total of 33% sera were positive^43^. Antigens of *Borrelia* and *Treponema* spirochetes often cross-react with each other. False positive results of serologic tests have been obtained in neurological patients with infections with other bacteria in the same group, for instance *T*. *pallidum*^42,44^. Therefore, our positive results of IgM and IgG ELISA tests were confirmed by Western Blot according to the European guidelines^45^ in order to exclude false positive results. Only half of positive ELISA results were confirmed in WB in HIV-positive patients. Accordingly, our data strongly recommends confirmatory testing in HIV-infected patients with ELISA positive Lyme screening.

*Borrelia* seroprevalence among blood donors in Europe does not exceed 10%^46–48^. In our study, the IgM and IgG seroplevalence in ELISA tests were rather similar (13.1% and 5.0%), and 23% (IgM) and 80% (IgG) of positive/doubtful results were confirmed in WB, respectively. All blood donors with anti-*Borrelia* IgG antibodies underwent borreliosis in the last 10 years. It is difficult to determine whether tested blood donors were spirochetemic at the time of blood giving, yet most of them declared multiple blood donation in the last decade. Till now, there are only limited and conflicting data on the number of spirochetes in the blood of spirochetemic Lyme disease patients^49,50^. However, as spirochetemia may occur in Lyme disease, the potential for transfusion-transmitted *B*. *burgdorferi* exists but has yet to be reported^51^. Ginzburg and co-authors have postulated that host-adapted *B*. *burgdorferi* survives poorly under blood storage conditions, particularly if the number of organisms per milliliter of human blood is low. We have not confirmed the presence of *B*. *burgdorferi* DNA in the blood of the tested group of donors (data not published) and, therefore, we conclude that the risk of transfusion-transmitted *B*. *burgdorferi* is rather negligible.

*Babesia* is transmitted primarily by ixodid ticks, although blood transfusion is another important cause of infection^52^. To date, babesiosis in HIV-infected individuals has been observed in the United States of America^53,54^, yet one case has been also recorded in Europe (Spain)^29^. In those cases, the phase of infection was determined as chronic since it lasted several months. Relapses of babesiosis occurred despite treatment which because of high parasitemia involved blood transfusions^54^. It is worth to note that recent studies have identified the first case of false-positive HIV serology that was associated with active babesiosis, yet after successful treatment of babesiosis, the positive HIV serology turned negative^55^. Currently, there is no data on *Babesia* seroprevalence in HIV-positive individuals. In our study, the IgM *B*. *microti* seroprevalence in HIV-positive patients was significantly higher than among blood donors whose estimated IgM and IgG seroprevalence (<2%) was comparable to that reported for *B*. *microti* among blood donors in Europe^56–58^. Nevertheless, IgG seroprevalence among HIV-positive patients and blood donors was similar, and we have not confirmed the presence of *Babesia* DNA in tested blood samples (data not published). Human babesiosis appears to be rare in Europe^9^, including Poland^11^, which is consistent with a low *I*. *ricinus* infection rate (1.6–2.8% in Poland, 0.5% in Slovakia, 0.7% in Switzerland)^4,59–61^.

Human ehrlichiosis and anaplasmosis are acute febrile tick-borne diseases caused by various species from the genera *Ehrlichia* and *Anaplasma*. *A*. *phagocytophilum*, causing human granulocytotropic anaplasmosis (HGA), and *Ehrlichia chaffeensis*, the etiologic agent of human monocytotropic ehrlichiosis (HME), are considered as an emerging zoonosis with clinical manifestations ranging from a mild febrile illness to a fulminant disease characterized by multi-organ system failure, especially in immunocompromised individuals^62^. HME and HGA have similar clinical presentations and both pathogens could be transmitted by transfusion or organ transplantation^63,64^. Till now, only one case of asymptomatic *A*. *phagocytopilum* infection in HIV-positive patient was confirmed^40^. In our study, HIV-infected patients and blood donors presented a similar rate of *A*. *phagocytophilum* seroprevalence (<3%). In Europe, among blood donors, antibodies to *A*. *phagocytophilum* vary from 5% in Belgium^65^ to 22% in Greece^66^. The low *A*. *phagocytophilum* seroprevalence rate found in our study is compatible with the small number of clinical cases identified so far^67^ as well as the low prevalence in *I*. *ricinus* ticks in Poland^5^.

In persons infected with HIV, ehrlichiosis caused by *E*. *chaffeensis* is often life-threatening^68^. However, the disease responds well to specific, antibiotic therapy, particularly when antibiotics are used at an early stage of infection^69^. The *E*. *chaffensis* seroprevalence in HIV-infected patients in the US was estimated at 1.7% and was similar to the one observed in healthy persons^70^. Nonetheless, in this group of patients the fatal false-negative results of serological test were reported. Accordingly, the incidence of ehrlichiosis in this population seems to be underestimated^68,69^. The severe pathology and multi-organ involvement in fatal ehrlichiosis that mimics toxic shock-like syndrome was observed in patients who are immunocompromised due to other infections, such as HIV or chemotherapy, and is thought to be related to dysregulation of the host immune response and immunopathologic mechanism that leads to tissue damage and multi-system organ failure^71,72^. In our study, the rate of IgG anti-*Ehrlichia* antibodies was almost three times higher in HIV-infected individuals than among blood donors. Although our positive patients have not declared clinical manifestation characteristic of tick-borne diseases, the diagnosis of HME in HIV-positive patients is complicated since the symptoms of HME often mimic typical findings commonly associated with HIV-infection^70^. Although in Europe the incidences of HGE are rather rare and the number of study about *E*. *chaffensis* infection in *I*. *ricinus* is limited, in HIV-positive patients ehrlichiosis should be considered as potential opportunistic infection.

Bacteria of *Bartonella* genus are distributed in diversified geographic areas and are transmitted by different arthropod vectors, such as sandflies, the human body louse, the cat flies and probably ticks. Contact with animals and vectors seems to be the most important mode of transmission – although the ability of *Bartonella* to survive in stored blood with the potential for transfusion-associated infection has been shown^73^. *Bartonella* infection presents varied clinical symptoms, mainly in HIV-infected patients whose bacillary anigiomatosis and hepatic peliosis are classically associated with AIDS^74^. Bartonellosis in this group of patients seems to be less frequent today, possibly because of earlier recognition of HIV serostatus and the lesser number of individuals with CD4 lymphocyte cell counts below 50 cells/mm^3^ ^75^. Our study demonstrated low *B*. *henselae* seroprevalence rate among HIV-infected patients and it was similar to that observed in a healthy population of blood donors (<4.5%). *Bartonella* seroreactivity rates varied between 2% and 30% in studies from Europe and the US with higher prevalence rates in intravenous drug users, homeless people, cat owners and veterinarians^76,77^. Among HIV-infected patients from Europe, anti-*Bartonella* antibodies were significantly higher than in our study and varied from 16% to 41% of individuals^78,79^. The likelihood of false-negative serological results are high in heavy immunocompromised patients with active *Bartonella* infection^80^. However, the majority of patients tested in our study have good immunological status – in 81% of tested patients, the level of lymphocyte CD4+ T cells was higher than 300/µl and most of the patients (82%) were on HAART. Furthermore, in contrast to the previous study where the prevalence of anti-*Bartonella* antibodies is inversely proportional to the number of CD4 lymphocytes^74,81^, we have not found significant association between the *Bartonella* serological status and lymphocyte CD4+ T cell level in HIV-positive patients. Our study presented only 19% of patients with low CD4 levels, which could significantly alter our results. Even though the rate of *Bartonella* infection in ticks in Poland is rather low (<2%^82^), the serological studies have proved that *B*. *henselae* and *B*. *quintana* are present and widely distributed in Poland in such specific risk groups as: alcoholics, veterinarians and cats’ owners^76^. Therefore, the further study determining the *Bartonella* prevalence among HIV-infected individuals, especially those with a fever of unknown origin, is needed.

Intracellular bacteria of the Rickettsiaceae family (spotted fever group (SFG) are responsible for tick-borne rickettsiosis. In humans, the syndrome manifests clinically mainly through rash, fever and ‘tache noire’, i.e. eschar developing at the site of the tick bite^83^. In HIV-positive patients, only isolated cases of Mediterranean spotted fever (MSF) have been reported and found to be caused primarily by *R*. *monacensis* and *R*. *conorii*^39^. Occassionally, MSF presentation is mimicked by primary HIV infection^84^. Till now, only few serological studies of *Rickettsia* infections among HIV-infected patients in Europe were performed. Nogueras et al^85^. have shown that seroprevalences of *R*. *typhi* and *R*. *felis* infections do not exceed 7% in this group of patients and were similar to those obtained in healthy subjects from the same region. In our study, the estimated the IgG seroprevalence of SFG *Rickettsia* was comparable and did not exceed 2% in both groups. Nevertheless, the SFG *Rickettsia* seroprevalence in Poland seems to be rather high in occupationally exposed populations (forestry and agricultural workers – 36%)^86^. Moreover, *Rickettsia* infection in ticks is high and varies from 4% to 53% depending on the tick species (*I*. *ricinus* vs. *D*. *reticulatus*, respectively)^5,87,88^. It is likely that, similarly to *Bartonella* infection, the serologic response in HIV-infected patients with good immunological status could be comparable to that of a healthy population. Therefore, the further study estimating the *Rickettsia* prevalence among HIV-infected individuals, especially with heavy immunosuppression, should be conducted.

In our study, no significant association was noted between the serological status of patients and their age, sex or lymphocyte CD4+ T cell level in HIV-positive patients. Such medical information about patients was available only for 65% individuals, and it was the primary limitation in our study. However, the previous studies, despite the fact that they possessed all the data, did not find any statistical relations between seropositivities and the assessed variables as well^43,79,85,89^.

Very few HIV-infected patients and blood donors were seropositive for two of the six studied pathogens. The single infection and coinfections were significantly more often noted in HIV-infected patients whose immunodeficiency significantly increases the risk of infection caused by pathogens. Simultaneous seropositivity for *A*. *phagocytophilum* and *E*. *chaffeensis* was observed in 59% of all coinfections. It is likely that cross-reactive antigens, shared by *Ehrlichia* and *Anaplasma* that induce cross-reactive antibodies, may affect the high rate of false-positive results in serological tests^62^. Due to this cross-reactivity among ehrlichial species, sera should be tested against both *E*. *chaffeensis* and *A*. *phagocytophilium* antigens when ascribing a specific etiology. Coinfections with *B*. *burgdorferi* and *B*. *microti* were the second most frequent combination noted only in HIV-infected patients. Dunn *et al*.^90^ observed that coinfection with *B*. *burgdorferi* and *B*. *microti* significantly increases *B*. *microti* parasitemia in mice and that larval ticks become infected with *B*. *microti* in greater numbers when fed on coinfected hosts. A possible explanation is that the host immune response to disseminating spirochetes is not restricted to the skin and may interfere with the splenic immune response, which is critical for the control and clearance of *B*. *microti* infection. Initial case reports suggested that concurrent Lyme disease and babesiosis are associated with severe illness^91^. Accordingly, concurrent babesiosis should be considered for any patient with Lyme disease who experiences more severe illness symptoms than expected, especially when the patient does not respond well to recommended antibiotic therapy.

In our study, the frequency of doubtful results for *Anaplasma*, *Ehlichia* and *Bartonella* was significantly higher in HIV-infected patients than in blood donors. The results of the previous studies have shown that the serological evaluation of the presence of IgG *Ehrlichia* antibodies in patients in a single sample is not sufficient to confirm ehrlichiosis. Non-specific reactions, which are more often observed in the group of patients infected with HIV, may result from the dysfunction of an immunological system, and they might be the consequence of the cross-reaction with other pathogens^92^. In consideration of the above, examination of many samples using concurrent serological and molecular tests might be crucial to confirm infection with the specific grade of pathogen (for example *Ehrlichia* or *Anaplasma*)^69,92^. Similarly, in the case of serological diagnostic of *Bartonella*, in patients infected with HIV, the non-specific or false positive results of serological tests are explained by the cross-reaction with the following pathogens: *Coxiella burnetti* or *Chlamydia trachomatis*^93,94^.

In conclusion, our study confirmed that the seroprevalence of tick-borne infections in HIV-infected patients is higher than that of the healthy population in Poland, however no association between serological status of patients and lymphocyte CD4+ T cell level has been observed. The frequency of tick-borne coinfections and doubtful results of serological tests seems to be higher in HIV-positive individuals. Although the advent of HAART had a considerable impact on the incidence of AIDS-associated opportunistic infections, the further studies of tick-borne infection in HIV-infected patients, particularly in patients who do not regain immunological function despite well controlled HIV replication on effective HAART, should be performed^95^. In this group of patients, tick-borne pathogens may cause chronic, debilitating opportunistic infection and even death. Thus, in clinical care of HIV-positive individuals, detailed history of tick bites in endemic tick areas should be collected via directed anamnesis. The low seroprevalence and negative results of molecular studies of tick-borne pathogens in blood donors (data not published) have suggested that the possibility of tick-borne diseases transmission with blood is rather negligible, which is consistent with the lack of reported cases of transfusion-transmitted tick-borne infections in Poland. Nevertheless, there is still a clear need to further such studies in order to maintain a balance between consideration of the real risk of disease transmission with blood and excessively restrictive approach that eliminates blood donors.

## Methods

### Selection and recruitment of patients/participants and serum samples

In 2016, serum samples were collected from 199 blood donors (representing the control group) who were diagnosed in AmerLab Ltd. Diagnostic Laboratory of Parasitic Diseases and Zoonotic Infections. Subjects with immunodeficiency were excluded. All participants signed informed consent and obtained a standardized, anonymous questionnaire to record data including age, gender, immunological status, place of residence, the history of tick bite, as well as previously diagnosed borreliosis or other tick-borne diseases. Blood samples obtained from blood donors were stored at room temperature and centrifuged immediately or within a maximum of 12 h after collection. Sera were frozen at −20 °C until further analysis.

The retrospective study was conducted on HIV-positive patients who did not have any known history of tick bite, nor any clinical manifestation characteristic for tick-borne diseases. In 2013, serum samples were collected from 227 patients routinely followed at the HIV Outpatients’ Clinic of the Hospital for Infectious Diseases in Warsaw.

The study protocol followed ethical guidelines of the 2013 Declaration of Helsinki and the study was approved by the Internal Review Board of the Warsaw Medical University (no. AKBE/24/16). Informed consent was obtained from all individual participants included in the study. All ethical approvals for the study have been obtained in accordance with the Polish regulations.

### Serological Tests

All serum samples (227 from HIV-positive patients and 199 from blood donors) were analyzed for the presence of antibodies against six tick-borne pathogens by using: (1) Borrelia IgM and IgG ELISA tests (Biomedica Laboratories, Vienna, Austria) for Borrelia burgdorferi s.l.; (2) Western Blot: recomLine Borrelia IgM and recomLine Borrelia IgG (Microgen, Neuried, Germany) for positive or doubtful results of Borrelia ELISA test for confirmation according to the European guidelines (Stanek *et al*. 2011); (3) Ehrlichia chaffeensis and Anaplasma phagocytophilum IFA IgM Antibody Kit (Fuller Laboratories, California, the USA; positive cut-off 1/512) for A. phagocytophilum i Ehrlichia spp.; (4) Babesia microti IFA IgM and IgG antibody kits (Fuller Laboratories, California, the USA; positive cut-off 1/128 for IgM and 1/512 for IgG) for Babesia spp.; (5) Spotted Fever Rickettsia IgG EIA antibody kit (Fuller Laboratories, California, the USA; the cut-off calibrator is set and the index value for each serum is derived. Indices from 0.9 to 1.1 absorbance units may be considered equivocal, while those above 1.1 are considered positive and those below 0.9 are considered negative) for Rickettsia spp.; (6) Bartonella henselae IFA Human IgM and IgG antibody Kit (Fuller Laboratories, California, the USA; positive cut-off 1/512) with the manufacturer’s interpretation criteria.

### Statistical analysis

Statistical analysis was performed using IBM SPSS Statistics v. 23.0 software. For the analysis of the results, doubtful serological results of tested pathogens were classified as negative. A descriptive analysis of the participants was included and calculations of seroprevalence rates for each pathogen were performed. Seroprevalence rates were compared with the tested group (HIV infected patients/blood donors), age, sex, lymphocyte T CD4+ level (HIV infected patients) using Maximum Likelihood techniques based on log-linear analysis of contingency tables (HILOGLINEAR).

### Ethics approval and consent to participate

The study protocol followed ethical guidelines of the 2013 Declaration of Helsinki. The study was approved by the Internal Review Board of the Warsaw Medical University (No. AKBE/24/16). Informed consent was obtained from all individual participants included in the study. All ethical approvals for the study have been obtained according to Polish regulations.

## Data Availability

The datasets generated during and/or analyzed during the current study are available from the corresponding author on reasonable request.
